# A Review on Recent Developments for Detection of Diabetic Retinopathy

**DOI:** 10.1155/2016/6838976

**Published:** 2016-09-29

**Authors:** Javeria Amin, Muhammad Sharif, Mussarat Yasmin

**Affiliations:** COMSATS Institute of Information Technology, Department of Computer Science, Wah 47040, Pakistan

## Abstract

Diabetic retinopathy is caused by the retinal micro vasculature which may be formed as a result of diabetes mellitus. Blindness may appear as a result of unchecked and severe cases of diabetic retinopathy. Manual inspection of fundus images to check morphological changes in microaneurysms, exudates, blood vessels, hemorrhages, and macula is a very time-consuming and tedious work. It can be made easily with the help of computer-aided system and intervariability for the observer. In this paper, several techniques for detecting microaneurysms, hemorrhages, and exudates are discussed for ultimate detection of nonproliferative diabetic retinopathy. Blood vessels detection techniques are also discussed for the diagnosis of proliferative diabetic retinopathy. Furthermore, the paper elaborates a discussion on the experiments accessed by authors for the detection of diabetic retinopathy. This work will be helpful for the researchers and technical persons who want to utilize the ongoing research in this area.

## 1. Introduction

**Figure 1:**
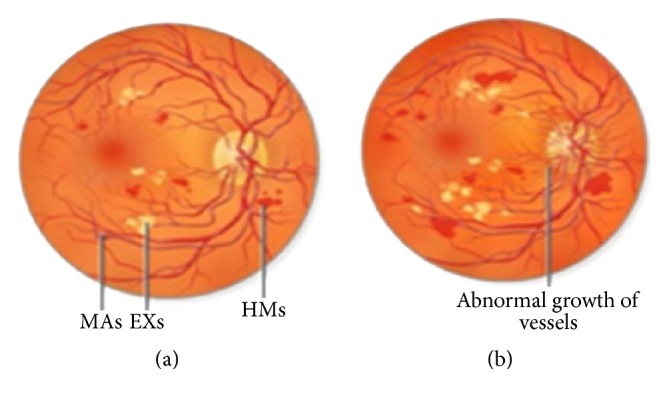
Stages of diabetic retinopathy. (a) Signs of NPDR. (b) Signs of PDR.

Diabetes is a very common disease worldwide. It serves as a most common cause of blindness for people having age less than 50 years. It is a systemic disease which is affecting up to 80 percent of people for more than 10 years. Many researchers acknowledged that 90 percent of diabetic patients could be saved from this disease through an early diagnose. A person having diabetes is more prone to the risk of diabetic retinopathy (DR) [1]. The blood supply towards all layers of retina is done through micro blood vessels which are susceptible to unrestrained blood sugar level. When a large amount of glucose or fructose gathers in blood, the vessels start crumbling because of insufficient distribution of oxygen to cells. Any blockage in these vessels leads to a severe eye injury. As a result, metabolic rate slows down and leads to structural abnormality in vessels which intern DR [2]. Microaneurysms are an earlier sign of DR. This disease brings changes in the size of blood vessels (swelling). The indications of DR include microaneurysms (MAs), exudates (EXs), and hemorrhages (HMs) as well as the abnormal growth of blood vessels. DR normally has two different stages named as proliferative DR (PDR) and nonproliferative DR (NPDR) [3]. Occurrence of NPDR is when blood vessels in retina are damaged and start leaking fluid onto it. As a result, retina becomes wet and swollen. Different signs of retinopathy exist at this stage, for example, HMs, MAs, EXs, and also interretinal micro vascular abnormalities (IRMA). PDR arises when new abnormal blood vessels appear in various areas of retina. It is a complex case of DR that may cause impaired vision [4]. DR is a progressive disease and its detection at an early stage is very crucial for saving a patient's vision; this requires regular screening. An automated screening system for DR can help in reducing the chances of complete blindness due to DR along with lowering the workload on ophthalmologists. For DR screening, a computer-aided diagnostic (CAD) system is developed for differentiating a retina with possible DR from a normal retina [5–7].  Figure 1 shows the symptoms for different stages of DR. The paper is organized as follows.  Section 2 describes different publicly available databases that are used for the detection of DR.  Section 3 mentions various performance measures that are taken into account for system evaluation.  Section 4 gives an overview of different methods being used for the detection of DR.  Section 5 highlights different techniques that are applied for the screening of DR.  Section 6 details retinal imaging techniques.  Section 7 focuses on methods for DR detection using CAD for the detection of DR different stages.

## 2. Publicly Available Databases

Images of retina are taken by a device called fundus camera. Retinal fundus images (RFI) is the name given to these images. This camera takes images of the internal surface of retina, posterior pole, macula, optic disc, and blood vessels. Image acquisition is a leading step for medical diagnosis. For research on medical fundus images, widely accessible resources are available as shown in Table 1. Some benchmark databases are openly available for the assessment of algorithms introduced for the computerized screening and analysis of DR. The purpose of databases is to check the strength of automatic screening of DR and then compare the results with current techniques. Seven datasets are available openly including DRIVE, STARE, DIARETDB, e-ophtha, HEI-MED, Retinopathy Online Challenge (ROC), and Messidor.

## 3. Performance Measures

For the diagnosis of DR in its early stage, the retinal image captured using some fundus camera needs to undergo preprocessing before applying further algorithms of image processing. Many preprocessing techniques such as contrast adjustment, average filtering, adaptive histogram equalization, homomorphic, and median filtering are applied on retinal images in the gold standard database. After an algorithm is applied to a retinal image, mean square error (MSE) and peak signal to noise ratio (PSNR) are further calculated to analyze the performance of an algorithm. PSNR is mostly denoted regarding logarithmic decibel value. A higher value of PSNR means that the processed image is of higher and better quality than the actual image. In medical treatment, the medical contribution data is frequently divided into two types: data where the disease is present and data where the disease is not diagnosed. The level of correctness of treatment is reviewed by the sensitivity and specificity measures. In medical research, fundus images which are common in DR are calculated via sensitivity and specificity of each image. The higher sensitivity and specificity values enhance the treatment. True positive (TP) denotes the total number of lesion pixels and true negative (TN) denotes the nonlesion pixels. False positive (FP) denotes the number of nonlesion pixels that are detected wrongly by the algorithm. Likewise, false negative (FN) indicates the number of lesion pixels that are not identified by the algorithm.  Table 2 shows performance metrics.

## 4. Methods for Detection of Diabetic Retinopathy

**Figure 2:**
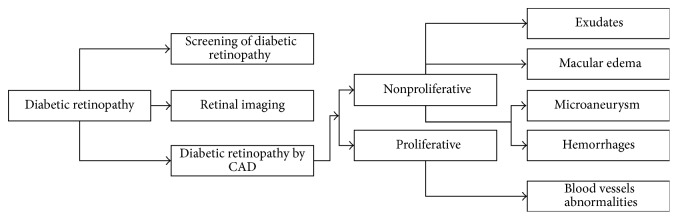
Methods for detection of diabetic retinopathy.

DR is a main reason of blindness. DR development is at different rates in different persons because of the two important vision pressuring difficulties: diabetic macular edema (DME) and proliferative retinopathy. That is why there is a huge demand for the latest technologies and methodologies to analyze DR efficiently and correctly in its early stage. Nowadays the research community has given many techniques shown in Figure 2 for early detection of DR that is conferred here.

## 5. Screening of Diabetic Retinopathy

Eye illnesses, for example, DR and DME, are the most widely recognized reasons for irreversible vision loss in people with diabetes. Just in the United States alone, health care and related costs identified with eye maladies are assessed at nearly $500 M. Besides, the prevalent cases of DR are relied upon to become exponentially influencing more than 300 M individuals around the world by 2025. Early discovery and treatment of DR and DME play a major role in preventing adverse effects such as blindness. Optical Coherence Tomography (OCT) imaging is ideal for uncovering DME on account of its extended axial resolution and extended retinal scan coverage, spatial domain optical coherence tomography (SD-OCT), revealed DME-related changes of the photoreceptors, external restricting film, Bruch's layer, and retinal pigment epithelium layer. Moreover, SD-OCT perceived related epiretinal layers, changes in the retinal decay, and vitreoretinal interface. The issues of automatic classification of SD-OCT information for automatically recognizable proof of patients with DME versus ordinary subjects are tended as well. The proposed technique depends on Local Binary Pattern (LBP) features to portray the texture of OCT pictures and contrast diverse LBP features extraction approaches with the process of single signature for the entire OCT volume. Test results with two datasets of separately 32 and 30 OCT volumes demonstrate that, regardless to utilizing low or high state representations, features obtained from LBP texture have profoundly discriminative power [14, 15]. A novel strategy is recommended that uses noncalibrated numerous perspective fundus pictures to dissect the swelling of macula. This development empowers the discovery and quantitative estimation of swollen zones by remote ophthalmologists. This ability is not accessible with a single image and inclined to error with a stereo fundus camera. Likewise, exhibit automatic algorithm to quantify features from the recreated image which are helpful in POC robotized conclusion of early macular edema, for example, before the presence of exudation. The proposed method is divided into three steps: initial, a preprocessing system at the same time enhances the dim microstructures and the procedure of macula and balances the image; second, all accessible perspectives are enlisted utilizing nonmorphological sparse features; at long last, a thick pyramidal optical flow is computed for each one of the images and statistically consolidated to build a naïve height map of macula. Results are demonstrated on three arrangements of synthetic images and two arrangements of real world images. These preparatory tests demonstrate the capacity to deduce a minimum swelling of 300 *μ*m and to correlate the recreation with the swollen area [16].

## 6. Retinal Imaging

Optical systems are enhanced by utilizing the method of adaptive optics (AO). It is accomplished by reducing the distortion in front wave effect. Diseased eye and normal eye can be evaluated for spacing, mosaic, and photography cell thickness through AO retinal imaging. With the help of retinal imaging, the inflammatory diseases are monitored for the posterior segment of fundus photography. Fundus fluorescein angiography (FA) and OCT are the most commonly used techniques for retinal imaging. These imaging modalities are helpful in the therapy of patients with inflammatory conditions for the correct diagnosis of posterior pole [17]. FA is an invasive technique which utilizes fluorescein color and OCT is a costly imaging strategy contrasted with fundus photos. Besides, utilizing fundus pictures DME can be automatically diagnosed. DME grading method is utilized for telescreening. Henceforth, DME grading method applied on fundus image modalities is reliable and inexpensive technique contrasted with biomicroscopy, OCT, and FA modalities [18]. En face regular or improved multicolor scanning laser ophthalmoscopy (SLO) images gained with HRA2 permit a superior perception of epiretinal films for preoperative assessment contrasted with SD-OCT based en face thickness guide or pseudoshading images obtained with Optomap while infrared or FA images are slightly suitable to portray epiretinal layers [19]. Three-dimensional regional statistics are used to detect disease macular area using OCT images. The proposed method is tested on five patients with retinal malady in OCT images which indicates 80.7% accuracy for the anomalous range [20].

## 7. Diabetic Retinopathy Detection by Computer-Aided Diagnostic System (CAD)

The objective of computer-aided diagnosis is to recognize DR and normal images in utilizing features like area of EXs, MAs, veins, texture, HMs, node points, and so forth [34, 35].

In order of two classes (DR and normal), for DR screening device was produced by (Usher et al. [23], Sinthanayothin et al. [36], Aptel et al. [21], Reza and Eswaran et al. [25], Gardner et al. [24], Kahai et al. [22], Osareh et al. [37], and Quellec et al. [38]) utilizing clinical components in particular EXs, veins, MA, Cotton Wool Spots (CWS), and HMs. Dark lesions were segmented out using moat operator and EXs were extricated using recursive region growing (RRGT) and adaptive intensity thresholding (AIT) [23]. Quellec et al. [38] have applied optimal filters to extricate MAs. The artificial neural network (ANN) [23, 24, 36] and Bayesian outline work [21] were used for classification. Their methods obtained specificity of 46.3% and sensitivity of 95.1% [23]. Support vector machine (SVM) kernel classifiers are employed in CAD framework to discover the absence or presence of DR. The anticipated CAD framework has managed the classification issues in DR [26]. Computer-aided diagnosis has assumed a fundamental part in medical industries [39]. To recognize DME, DR, and essential injury, an algorithm in view of examination of mechanized fundus photo was proposed. While screening for DR, this algorithm is an enhanced substitution for the fundus photo manual examination which expends a considerable measure of time. If this framework is operated by the doctors, it will allow significant time saving hence enhancing the time spent on a mass screening program [27]. In medicinal imaging, contextual information performs an important role. Bright lesions detection and discrimination has been performed through contextual information in fundus image modalities. Diagnosis of coronary calcifications and hard EXs in CT scan is performed through this contextual information. In the spatial relation, high-level contextual features are used to explain the context. Contextual CAD framework is an outflanked loom when contrasted with local CAD framework [40]. Several authors have suggested CAD methods for the detection of different stages (NPDR and PDR) of DR that are encountered and described in this section.  Table 3 summarizes these CAD methods.

### 7.1. Segmentation and Localization of Optic Disc

**Figure 3:**
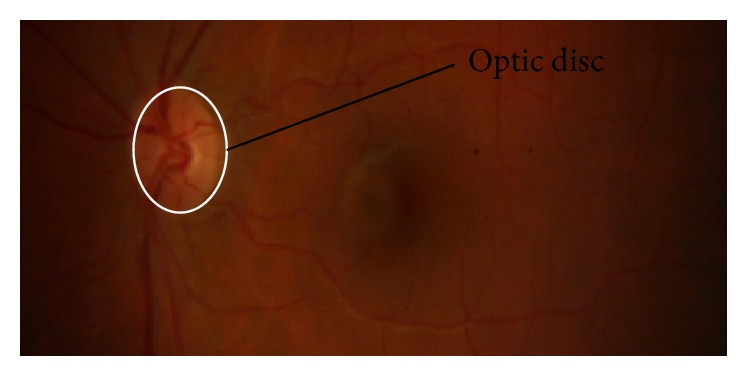
Optic disc in fundus image.

The optic disc (OD) is a round region in the back of eye where retinal nerve fibers gather to frame the optic nerve. OD is sometimes called optic nerve head (ONH) since it is the leader of optic nerve as it enters eye from the brain. It is found marginally to the nasal side of globe. OD is known as the blind side since it contains no photoreceptors. In this way, any light centered on OD can neither be changed over into sensory impulses nor sent to the brain for elucidation.  Figure 3 shows OD in a fundus image. For the detection of DR, first step is segmentation and localization of OD because its color, intensity, and contrast are the same as other features of retinal images. Many authors have suggested CAD methods for the segmentation and localization of OD that are described in Table 4.

For automatically finding ONH, the strategy of Gabor filters in fundus image is used as the ONH center which is close to the focal point of retinal vessels and the phase portrait analysis [60, 61]. New method is proposed for the localization and detection of OD. The proposed technique gives better results [62]. Histogram matching method is used for OD localization [63]. Morphological filtering strategies and watershed transformation are used for OD detection and localization. The proposed method has been tested on 30 color fundus images. Subsequently achieved mean predictive values and sensitivity were 92.4% and 92.8%, respectively [64]. The proposed method is used for localizing the OD center that depends on corners and bifurcations attained with Harris corner detector. The achievement rate is 87.65% for STARE, 97.5% for DRIVE, and 97.8% for local dataset, respectively [65]. Macula centers and OD are detected using radial symmetry method [66, 67]. DR is diagnosed by using the method of retinal extraction. In automatic screening, OD is detected at very low cost. Locating the center of OD is difficult because the color, brightness, and contrast are similar to CWS and EXs [68]. Boundary tracing technique is used for locating the OD boundary [69]. Multilevel 2D wavelet decomposition method is used for the localization of OD. HRF database is utilized for the assessment of results with Receiver Operating Characteristic (ROC) curve and 95% accuracy is achieved for the localization of OD [29]. For OD detection and location, a new method is proposed in the light of histogram approaches and clustering. The method is evaluated with Messidor dataset which demonstrates that it can localize the OD accurately even in blurred images [30]. A line operator is proposed to capture round brilliance structure which assesses the image shine variety along various line segments of particular orientations that go through every image pixel of retina. The orientation of line segment with base/greatest variety has a particular example that can be utilized to find the OD precisely. Suggested line operator is tolerant to a normal OD identification, various sorts of lesions in retinal imaging modalities with an achievement of 97.4% accuracy [31]. A novel algorithm is proposed for the segmentation of OD in images of retina while making use of Atanassov Intuitionistic Fluffy Histon (AIFSH) based segmentation method. Columnwise neighborhood operation and pixel intensity of OD are utilized to find and separate the OD [32]. A novel method is proposed depending on a symmetry transform and in painting which is applied very competitively in tests with a publicly available and local dataset [33]. A summarization of different OD detection methods is shown in Table 4.

### 7.2. Segmentation of Soft and Hard Exudates (NPDR)

EXs are one of the primary signs of DR which can be prevented with an early screening process. Some existing work for the detection of EXs is described below.

**Figure 4:**
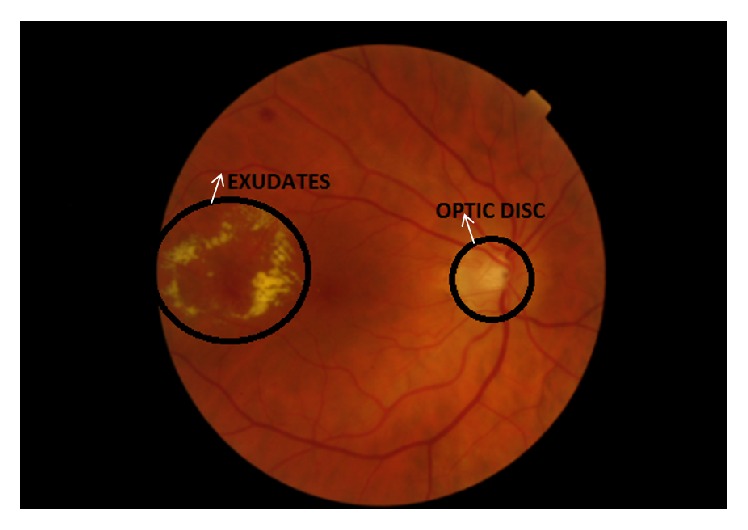
Fundus image with exudates.

A novel approach is proposed to detect DR automatically from digital fundus images. Digital images play a significant role in the identification of DR by calculating various color spaces in segmented region. DR is detected by fuzzy set using fuzzy logic reasoning [86]. In developed countries, visual impairment is a significant reason of DR. The disease can be detected to have the assistance of fundus pictures identified with DR lesions. For this reason, a method is proposed using a neural network. The proposed methodology is completely automatic after the configuration of classifiers [87]. EXs can be detected using multispace clustering method [41, 50].  Figure 4 presents a fundus image with the symptoms of EXs.

The proposed method is reliable for the identification of EXs in retinal images. Morphological operators and adaptive thresholding method are utilized in the computation of noise map distribution. Contrast changes and nonuniform illumination method are used for the detection of correct EXs [42]. The proposed method is used for accurate segmentation of EXs [88]. For the diagnosis of bright lesions *K*-means clustering method is applied [89]. A novel method is proposed utilized neural network to minimize DR with high accuracy [43]. Both KNNFP and WKNNFP classifiers are used to detect the EXs but WKNNFP shows better results as compared to KNNFP [44]. Bright lesion can be detected using Gabor filter [90]. Mathematical morphology algorithms and *K*-means are utilized for the identification of bright lesions [45]. EXs can be recognized with the help of gray level variation and their contours are determined using the morphological reconstruction methods [91]. Haar wavelets transform is used for the hard EXs segmentation followed by *K*-nearest neighbor classification method. The proposed method is tested on four databases of fundus images; among them, obtained sensitivity is 37.14%, 21.87%, 12.50%, and 25.47% for MISP (Medical Image and Signal Processing Research Center), DIRETDB0, DIREDB1, and STARE database, respectively. Likewise the specificity is 0% for MISP and 1% for remaining databases [46]. The suggested technique utilizes several image processing methods including image thresholding and, median filtering with an aim to discover hard EXs. The suggested technique showed specificity of 96.85% and sensitivity of 97.25% [2]. A novel technique is proposed for the discovery of bright lesions in color retinal images. Intellectual decision support system is utilized for DR detection. The texture and color features are applied for distinguishing between non-EXs and EXs pixels. Initially, edge discovery and morphological process are used for the segmentation of OD. Secondly, for attaining texture features from the area of retina, color and laws texture energy measures are performed. Afterwards, an intelligent classifier Fuzzy SVM has been utilized to discover pathological regions in color fundus images [47]. The EXs discovery technique comprises two stages: fine and rough EXs segmentation. Rough segmentation has been applied using columnwise neighborhoods and morphology operation whereas morphological reconstruction method is practical for fine segmentation. The suggested technique used retinal image database from Malaysia, Sungai Buloh Hospital. Organized with other appropriate retinal features, extraction and classification technique, this segmentation technique can form the basis of an easy and fast diagnostic support tool for DR which will provide a great benefit regarding better access to mass screening people for risk or existence of diabetes [48]. The new technique is proposed for automatic discovery of human fundus image by the submission of digital image processing. Circular bit plane slicing and Hough transform are applied for OD localization in the proposed technique whereas, for the extraction of EXs, morphological operations are used. The suggested technique is a novel method. It has a sensitivity of 93.62% and an accuracy of 88% [49]. Bag of Words algorithm is used to make a system which plays the role of both the case based reasoning (CBR) system and decision support system (DSS) to solve the problem of bright lesion segmentation [51]. For the segmentation of EXs, dynamic region growing method is proposed. The method is tested on several images of retina and the results demonstrate that the method does better than the earlier suggested techniques [52]. Novel measurable atlas based technique is proposed for the segmentation of EXs. Any test fundus picture is initially distorted on Atlas coordinate and after that a distance map is obtained with the mean atlas picture. An analysis of openly accessible HEI-MED dataset shows great presentation of the technique. On the FROC curve, 35% nonlesion localization fraction and 82.5% localization fraction are obtained. The technique is additionally contrasted with couple of latest reference strategies [92]. Hard EXs can be characterized by the DME. Novel features set based on color and wavelet decomposition are used for DME detection. Classifier is trained using these features to automatically analyze DME through the occurrence of exudation. Another freely accessible HEI-MED database is there with ground-truth information containing 169 patients. Our calculation acquired an AUC somewhere around 0.88 and 0.94 relying upon the dataset/features utilized [93].

A brief summary of methods for the detection of EXs is given in Table 5.

### 7.3. Macular Edema Detection (NPDR)

**Figure 5:**
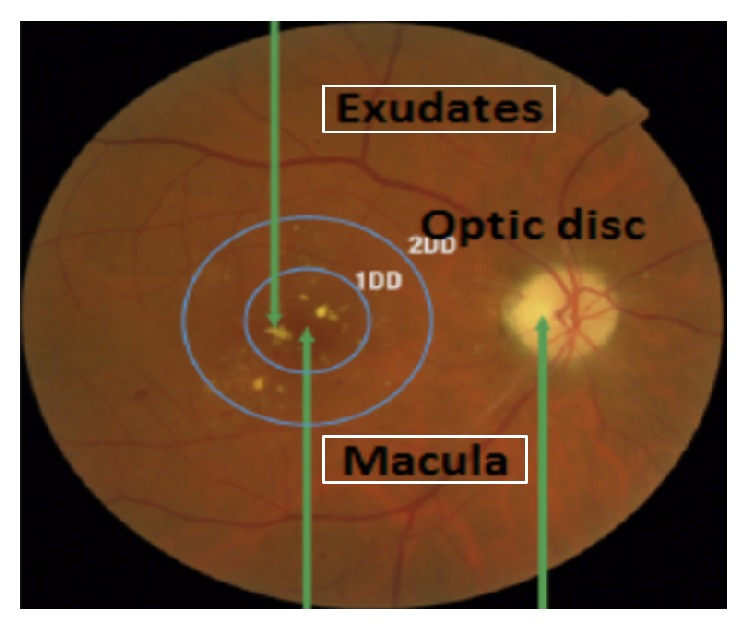
Macula exudates and optic disc in fundus image.

Macula is the center point of vision where light is focused. In a fundus image, macula appears at the center of retina with a blackish color due to an excess of melanin in its composition. It interprets the images and sends them to brain. Its size is 3 mm. If lipids and proteins start accumulating near or on the macula, it can become as dark lesions; these are called macular edema.  Figure 5 shows EXs, macula, and OD in a fundus image. Many researchers have proposed CAD systems for the detection of DME that are described in Figure 5.

DME is a fundamental reason of visual harm. The pathogenesis of macular edema seems to be multifactorial. Laser photocoagulation is the standard of care for macular edema [94, 95]. Surgical therapy and pharmacologic method are used to handle DME in diabetic patients [96]. The qualitative OCT and clinical examinations are required to be performed on monthly basis to check the anti-VEGF (vascular endothelial growth factor) for the maximization ratio of visual acuity gain along with the requirement of some injections [97, 98]. The treatment by injections is safe over two years and effective for DME. DME treatments include VEGF drugs. There is no damage to retina by the Micropulse Diode Laser (MPL) treatment. Retinal pigment epithelium (RPE) is used for different MPL stimulation. ETDRS photocoagulation group is compared with laser group and found that retinal sensitivity has been increased [99, 100]. Gaussian mixture model (GMM) based classifier and detailed feature set are utilized for perfect and accurate detection of macula; this nominated/submitted system consists of a novel method. Both SVM and ensemble of GMM classifiers are used for an accurate detection of EXs which ultimately leads to perfect classification of retinal image in different stages of macular edema. The same system has got mean value of 95.9%, 97.3%, and 96.8% for specificity, sensitivity, and accuracy, respectively [101]. Raja and Ravichandran [102] explain a way to autolocalize the fovea center in retinal fundus images. This method is specifically based on mathematical morphology beside the information of other anatomic structures such as blood vessels and OD. Firstly, the vascular structure and OD center are extracted and then morphological operations are employed on the gray scale image of green channel for fovea candidates' selection. The candidates satisfying area, density, and distance criteria are considered for the final stage. And there, the candidate having lesser vessel pixels is selected as fovea region. The method was evaluated on two publicly available STARE and DRIVE databases. It was able to obtain 100% of fovea localization accuracy on DRIVE database with 2.88 seconds average computation time.

### 7.4. Microaneurysms Detection (NPDR)

**Figure 6:**
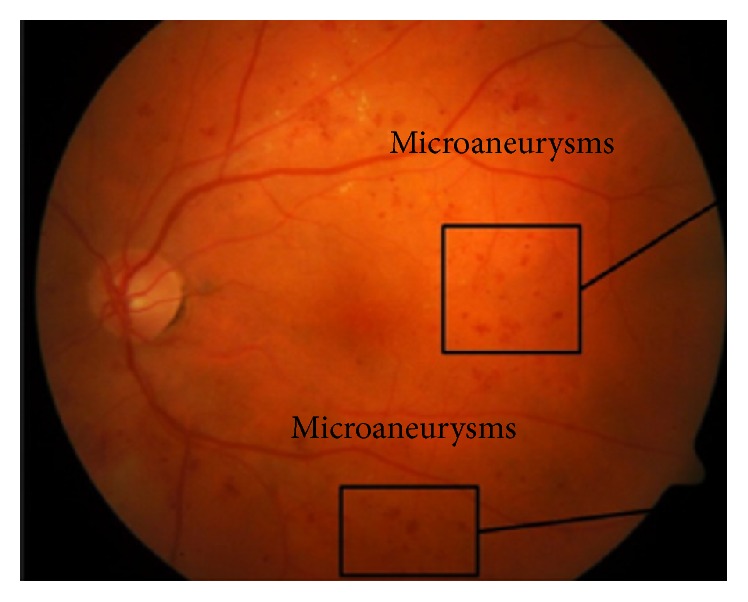
Microaneurysms in retinal image.

An early symptom of DR is MAs; MAs diagnosis is significant in early detection of DR. MAs are the major symptom of NPDR and are initiated by the principal dilatations of thin blood vessels.  Figure 6 shows MAs in retinal image. Several proposed CAD systems for the detection of MAs are discussed in Figure 6.

MAs are of small dimensions, nearly red in color, and round [103]. Dynamic thresholding and multiscale correlation filtering (MSCF) method are used for MAs detection. The proposed method contains two levels, coarse level (MAs candidate detection) and fine level (true MAs classification). The method was tested on two publicly available datasets namely ROC and DIARETDB1 databases [104]. *K*-nearest neighbor classifier (*K*NN) is used for the detection of MAs [105]. Morphological operators are applied for MAs detection in fundus images. The method obtained 99.98% accuracy, 81.61% sensitivity, 63.76% precision, and 99.99% specificity [53]. An ensemble-based framework is nominated to improve MAs detection [54]. The proposed method comprises two stages. In the first stage, preprocessing is performed using fractal analysis of retinal vascular structure. The principle stage contains picture preprocessing and fractal analysis of retinal vascular structure. If fractal examination distinguishes an abnormal image from a normal one, this enhances the effectiveness of a computerized screening program. Identification of MAs distinctive shape as an abnormal retinal picture through morphological reconstruction methods and canny edge detection is an objective of second stage. The proposed calculation has been performed on an arrangement of 89 fundus pictures from the accessible database. The applied method achieves operating sensitivity and highest specificity as 89.5% and 82.1%, respectively [55]. Contrast enhancement approach with Singular Value Decomposition (SVD) method is applied to fundus images. After it, Hessian-based candidate selection method is used for the detection of MAs. For every candidate region, intensity normalized radon transform and robust low-level blob descriptors are known as SURF and take out to characterize MAs candidate regions. Then, classification is performed along with SVM classifier which has been trained with ten manually annotated training images. Presentation of complete system is estimated on ROC database [56]. Contrast Limited Adaptive Histogram Equalization (CLAHE) method is used to enhance contrast of images for the detection of lesions [57]. The localization and detection of MAs are based on three steps: candidates' selection, segmentation of MAs, and a final performance evaluation. New radon cliff operator is proposed which is an actual contribution to the field [58]. The dynamic multiparameter template (DMPT) matching scheme is applied for the detection of MAs that is more accurate as compared to conventional schemes [59].

A summary of MAs detection methods is presented in Table 6.

### 7.5. Hemorrhages (NPDR)

**Figure 7:**
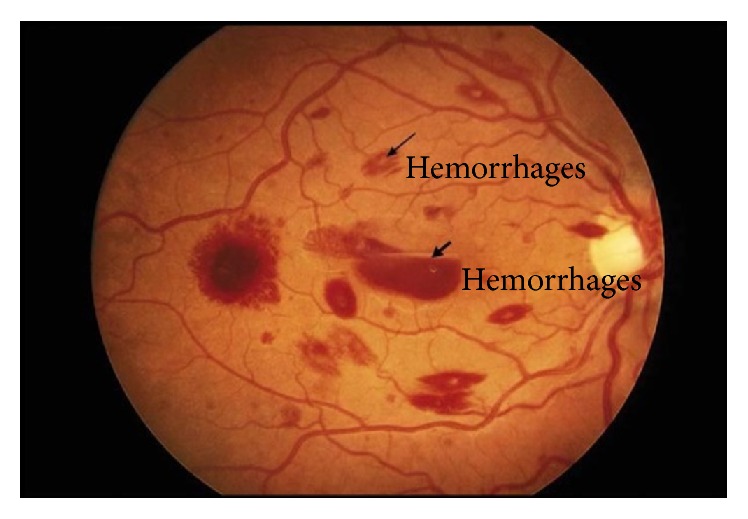
Hemorrhages in fundus image.

HMs occurs when blood outflows from retinal vessels.  Figure 7 shows HMs in a fundus image. Many authors suggested CAD systems for detection of HMs which are listed and discussed in Figure 7.

The proposed technique used HSV color space by nonlinear curve for changing the brightness of fundus images. Brown regions are highlighted using gamma correction in each blue, green, and red bit image. Then, histogram of each blue, green, and red bit image was extended. Brown regions represented HMs and blood vessels. Then, density estimation is applied to find brown regions. False positives were eliminated via 45-feature examination. The proposed method is tested on 125 fundus images along with 90 normal images and 35 images with HMs [70]. In fundus image, a novel splat feature division method is proposed for retinal HMs detection. A new method is proposed for the preprocessing and false positive elimination. A classifier is prepared with splat-based skilled observations and expanded on openly accessible Messidor dataset [71]. Automated Decision Support System (DSS) is developed for the detection of HMs and MAs in fundus images. The severity level of DR is determined by the location and number of HMs and MAs. The method is tested on 98 fundus images. Correspondingly, experimental outcomes show that 87.53% and 84.31% sensitivity and 95.08% and 93.63% specificity values were obtained by the proposed system for HMs and MAs discovery [72, 73]. Sudha and Thirupurasundari [74] suggested an automated system to discover DR from retinal images. In this method after preprocessing, texture features are taken out from retinal images to discover abnormal images. Afterwards, the abnormal images are treated to localize and classify the problem of HMs and EXs. Dynamic thresholding method is used for the detection of HMs in retinal images. Experimental results show that the HMs are discovered with good accurateness in retinal images [75]. A novel hybrid classifier is proposed for the discovery of retinal lesions. The suggested method comprises preprocessing, taking out of feature set formulation, and classification of candidate lesions. The system is measured using standard fundus image databases with the support of performance parameters known as specificity, accuracy, sensitivity, and ROC curves for statistical implementation [76]. For HMs detection concentration is on the examination of texture micro-patterns of regions of interest (ROIs) which are concerned areas in an image of retina. Texture micro-structures of ROIs are examined via LBP for their explanation. Lastly, SVM is applied to indicate whether an ROI consists of HMs or not [77]. A brief overview of commonly used methods for HMs detection is given in Table 7.

### 7.6. Blood Vessel Detection (PDR)

**Figure 8:**
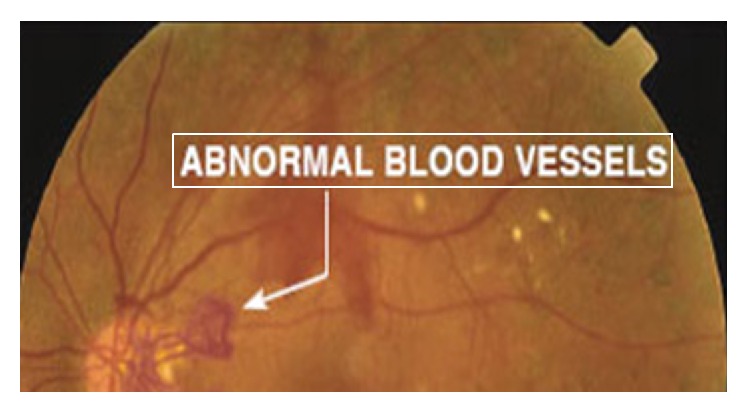
Abnormal blood vessels.

PDR occurs when there is formation of some new abnormal blood vessels in different regions of retina. It is an advanced stage of DR and may lead to complete blindness. Hence, for PDR diagnosis, blood vessels detection is an important task.  Figure 8 shows abnormal blood vessels. Some CAD methods for the detection of blood vessels are described in Figure 8.

DR is described against the classification and detection of changes, in time series, as presented in fully automated approach [28, 106]. This method consists of the following steps: (1) illumination from instrument and patient visit, (2) dust particle imaging, (3) training data, and (4) alignment and segmentation error of retinal vasculature, fovea, and OD. Pathologies are extracted automatically and their robustness is achieved by an algorithm. The technique is presented for the bifurcation and geometric model without the intervention of user [107]. For blood vessels segmentation, the proposed method applied subpixel root MSE with the adoption of preprocessing, Multilevel Thresholding, H-maxima transformation, and Binarization. From this technique accuracy rate of 96.5% is obtained [108]. A novel method is proposed utilizing Gumbel probability distribution function as its kernel and matched filter. The method gives accurate results for blood vessels segmentation [109]. Similarly, the methods of branches filtering and Max-Tree representation are utilized for the segmentation of vessels with 93.95% success rate [110]. Fuzzy *K*-median and length filter (FKMED) and matched filter methods are applied for blood vessels segmentation in fundus images. The method achieved sensitivity and specificity as 79.31% and 96.43% [111]. Another derived methodology is Gaussian Intensity Distribution (GID) model utilized for blood vessels segmentation [112]. Morphology method and canny algorithm are also used for blood vessels segmentation. The proposed method is tested on DRIVE dataset [113]. The authors made use of matched filter and threshold probing for blood vessels segmentation. Grid division, nonuniform enhancement, and optimal multithreshold method are used for blood vessels segmentation [114]. Analysis of DR on edge detection method is based on content-based image retrieval (CBIR) framework. Preprocessing methods are subjected to normal and abnormal images of retina to enhance the edge information. Kirsch template and canny edge are two different methods that are practical for blood vessels segmentation in the diagnosis of retinal abnormalities. The kirsch edges are useful in CBIR system [115, 116]. Fraz et al. [78] introduced an ensemble system of boosted decision trees where bagged is used as feature vectors based on orientation analysis of gradient vector field, Gabor filter responses, line strength measures, and morphological transformation. To handle the pathological retinal image, the feature vector encodes information. For the segmentation of blood vessels, an automatic method is suggested. The submitted method is based on the fact that, by varying the length of a basic line detector, line detectors at continuously changing scales are achieved. In order to perform final segmentation for every image of retina, line responses are linearly combined at varying scales so that the strength and drawbacks elimination of each line detector are maintained. Both quantitative performance and qualitative performance of this method were evaluated on three publicly available DRIVE, REVIEW, and STARE datasets [79]. For pixel classification based method to segment blood vessels of retina, linear discriminant analysis is described in detail. In this method, vesselness measure of a pixel is defined by Gabor filter responses and the feature vector is comprised of a modified multiscale line operator [80]. A concise methodology is introduced for segmenting the retinal vasculature in color fundus images [81]. Lattice Neural Networks with Dendritic Processing (LNNDP) method is used to solve this problem [82]. Pixel classification is done by a neural network (NN) scheme [83]. Multidirectional morphological top-hat transform with rotating structuring elements and enhanced multiscale line detector are utilized for blood vessels detection [84]. Morphological operators are used for detecting blood vessel tree. In retinal fundus images, identification of abnormal spots is done more accurately after vessel detection. Experimental results are taken from Nikookari database which is consisting of 40 fundus images. The method achieved 85.82% average sensitivity and 99.98% average specificity [85].

A summary of blood vessels detection methods is presented in Table 8.

## 8. Future Directions

In medical image processing, automatic diagnosis of DR from digital fundus images has been a dynamic research for a long time. The research interest is justified by considerable reductions in health care costs and tremendous potential for new products in medical industry. There are certain areas in this field that need improvement such as the determination of OD boundary which is tough in two-dimensional retinal images because of blur edges. A difficult task to perform is blood vessels extraction. Every subject has different ONH structure. Therefore, there is not a single technique that can cover all these problems. There is still need to propose more efficient algorithms for the identification of DR detection related structure and retinal changes. Because of the expanding predominance of diabetes mellitus, demand for diabetic retinopathy screening stages is steeply expanding. Early location and treatment of DR are essential public health intercessions that can incredibly diminish the probability of vision loss. Current DR screening programs commonly utilize retinal fundus photography which depends on talented readers for manual DR evaluation. However, this is labor-intensive and suffers from inconsistency across sites. Hence, there has been the latest proliferation of computerized retinal image investigation programming that might mitigate this weight cost viably. Moreover, current screening programs given 2-dimensional fundus photography do not properly screen for DME. OCT is turning out to be progressively perceived as a reference standard for DME evaluation and can give an economical solution for enhancing DME discovery in vast scale DR screening programs. Current screening systems are additionally not able to picture the peripheral retina and require pharmacological pupil dilation; ultra-wide field imaging and confocal examining laser ophthalmoscopy which addresses these disadvantages have excessive potential.

## 9. Conclusion

Diabetic retinopathy cannot be cured. To prevent vision loss, laser analysis (photocoagulation) is usually very effective if it is done before it adversely harms the retina. Provided that the stern destruction of retina has not been done, vision can be improved by the surgical elimination of vitreous gel (vitrectomy). In proliferative diabetic retinopathy, at times, an anti-inflammatory medicine or antivascular endothelial growth factor medication injection can help in the new blood vessels contraction process. Since symptoms cannot build up until the disease turns into the stern, initial discovery via regular screening is essential. Nonproliferative diabetic retinopathy contains early indications of DR and it is extremely critical to recognize and analyze DR at its initial stages. If a person with diabetes gets legitimate eye mind consistently and treatment when fundamental, DR will once in a while cause all out blindness. In this study of DR, a large portion of work is done to discover hemorrhages, microaneurysms and exudates, diabetic macular edema, and abnormal new blood vessels as they are indications of the vicinity of retinopathy in fundus images. This study helps in the detection of retinopathy at an early stage; timely treatment of this disease will prevent permanent vision loss. The paper discussed experiments done by authors for the detection of diabetic retinopathy. This work will be useful for technical persons and researchers who need to use the ongoing research in this area.

## Figures and Tables

**Table 1:** Databases for fundus images.

Name	Image acquisition	Number of images	Resolution	Uses
DRIVE [8]	3-CCD camera with 45-fold view	20 color fundus testing images. 20 color fundus training images.	768 × 584	Exudates, hemorrhages, microaneurysms, and abnormal blood vessels detection

Image-Ret (DIARETB0, DIARETB1) [9]	50-fold view	DIARETB0: total 130 images in which 20 images are normal and 110 with DR. DIARETB1: total 89 images: five images are normal and 84 images with DR.	1500 × 1152	Exudates, hemorrhages, microaneurysms, and abnormal blood vessels detection

Messidor [10]	3CCD camera at 45-fold view	1200 images.	1440 × 960, 2240 × 1488, and 2304 × 1536	Exudates, hemorrhages, microaneurysms, and abnormal blood vessels detection

Retinopathy Online Challenge [11]	Canon CR5-45-NM camera at 45-fold view	100 digital fundus images.	768 × 576, 1058 × 1061, and 1389 × 1383	Microaneurysms detection

e-ophtha_EX [12]	OPHDIAT© Tele-medical network	It contains 47 images with exudates and 35 images with no lesion.	2048 × 1360	Exudates detection

e-ophtha_MA [12]	OPHDIAT© Tele-medical network	It contains 148 images with microaneurysms or small hemorrhages and 233 images with no lesion.	2048 × 1360	Microaneurysms detection

STARE [13]	Top con TRV 50 fundus camera at 35-fold view	400 images.	605 × 700	Exudates, hemorrhages, microaneurysms, and abnormal blood vessels detection

HEI-MED [7]	Zeiss Visucam PRO fundus camera at 45-fold view	169 images in which 115 images are abnormal and 54 images are healthy.	2196 × 1958	Exudates detection

**Table 2:** Performance metrics.

Measures	Description	
Peak signal to noise ratio (PSNR)	20 log_10_⁡(MAX_*I*_) − 10 log_10_⁡(MSE)	Measures quality of image.

Sensitivity or true positive rate (TPR)	$TPR=\frac{TP}{P}=\frac{TP}{TP+FN}$	Measures the ratio between TP and FN.

False positive rate (FPR)	$FPR=\frac{FP}{N}=\frac{FP}{FP+TN}=(1-SPC)$	Measures the ratio between FP and TN.

False negative rate (FNR)	$FNR=\frac{FN}{TP+FN}=(1-TPR)$	Measures the ratio between FN and TP.

Specificity (SPC) or true negative rate (TNR)	$SPC=\frac{TN}{N}=\frac{TN}{TN+FP}$	Measures the ratio between TN and FP.

Accuracy (ACC)	$ACC=\frac{\left(TP+TN\right)}{TP+FP+FN+TN}$	The degree to which the result of a measurement, calculation, or specification confirms the correct value or a standard.

Area under curve (AUC)	*A* = ∫_∞_^−∞^TPR(*T*)FPR′(*T*)d*T*	How much system is sensitive to detect the desired output?

**Table 3:** CAD methods for diagnosis of DR.

Algorithm	Image processing techniques	Database	Color space	Sensitivity	Specificity	Accuracy/AUC	Lesions detection
Aptel et al. [21]	Single-field nonmydriatic; single-field mydriatic; three-field nonmydriatic; three-field mydriatic	79 patients (158 eyes)	Gray scale	77%, 90%, 92%, 97%	99%, 98%, 97%, 98%	0.82, 0.90, 0.90, 0.95	Both NPDR and PDR

Kahai et al. [22]	Decision support system (DSS)	143 images Louisiana State University Eye Center	Gray scale	100%	67%	—	NPDR

Usher et al. [23]	Neural network	1273 consecutive patients, St. Thomas Hospital	Gray scale	94.8%	—	—	NPDR

Gardner et al. [24]	Back propagation neural network	200 diabetic and 101 normal images of private hospital	Gray scale	88.4%	83.5%	—	Both NPDR and PDR

Reza and Eswaran [25]	Rule based classifier	STARE	Green channel	97.2%	100%	97%	NPDR

Annie Grace Vimala and Kajamohideen [26]	Cost-effective computer-aided diagnostic system	Private eye Hospital	HSV	91.6%	90.5%	91.2%	NPDR

Dupas et al. [27]	Automated fundus photograph analysis algorithms	Messidor	Gray scale	83.9%	72.7%	—	NPDR

Ashraf et al. [77]	Local Binary Pattern, SVM	DIARETB1	Green channel	87.48%	85.99%	86.15%/0.87	NPDR

**Table 4:** Different methods for detection of optic disc.

Algorithm	Image processing techniques	Database	Color space	Accuracy
Rathod et al. [29]	Multilevel 2D wavelet, Histogram Equalization	HRF	Green channel	95%

Sekar and Nagarajan [30]	OD localization based on clustering and histogram approaches	Messidor	RGB	99.58%

Lu and Lim [31]	Circular transformation line operator	DIARETDB0 DIARETDB1 DRIVE STARE	CIELAB	97.4%

Acharya et al. [32]	Otsu, Gradient vector flow (GVF) snake, Atanassov Intuitionistic Fuzzy Histon (AIFSH)	Kasturba Medical College	Gray scale	100%

Trucco et al. [33]	Binary vessel masks are developed within VAMPIRE software	HRIS (High-Resolution Image Set) VDIS (Vascular Disease Image Set)	Gray scale	95.7% 92.1%

**Table 5:** Different methods for detection of exudates.

Algorithm	Image processing techniques	Database	Color space	Sensitivity	Specificity	Accuracy
Ram and Sivaswamy [41]	Clustering-based method and color space features	DIARETDB1	RGB, CIE *L* ^*∗*^ *u* ^*∗*^ *v* ^*∗*^, HSV, HIS	71.96%	—	89.7%

Soares et al. [42]	Morphological operators and adaptive thresholding	DIARETDB1	Green channel	97.49%	99.95%	99.91%

Jayakumari and Santhanam [43]	Energy minimization method using echo state neural network	Private Hospital	—	90%	—	—

Karegowda et al. [44]	KNNFP and WKNNFP classifiers	DIARETDB1	HIS	—	—	97.50% WKNNFP 96.67% KNNFP

Amel et al. [45]	Combine the *K*-means clustering algorithm and mathematical morphology	Ophthalmologic Images	CIELab	95.92%	99.78%	99.70%

Rokade and Manza [46]	Haar wavelets transformation, KNN classifier	MISP DIRETDB0, DIRETDB1, STARE	Green channel	37.14%, 21.87%, 12.50%, 25.47%	—	—

Kayal and Banerjee [2]	Median filtering, image thresholding	DIARETDB0 DIARETDB1	Gray scale	97.25%	96.85%	—

Jaya et al. [47]	Morphological operations, Circular Hough transform, Fuzzy support vector machine	Private Hospital	—	94.1%	90.0%	—

Rozlan et al. [48]	Morphology operation, columnwise neighborhoods operation	Sungai Buloh Hospital	Green channel	—	—	60%

Soman and Ravi [49]	Circular Hough transform and bit plane slicing, morphological operations	Standard Diabetic Retinopathy	Green channel	0.9362	—	88%

Annunziata et al. [50]	Multiple scale Hessian approach	STARE HRF	Green channel	—	—	95.62% 95.81%

Van Grinsven et al. [51]	Bag of Words approach	Messidor EUGENDA	HSV, YCbCr	—	—	0.90 AUC

Kaur and Mittal [52]	Dynamic region growing method	SGHS hospital	Gray scale	—	—	98.65%

**Table 6:** Different methods for detection of microaneurysms.

Algorithm	Image processing techniques	Database	Color space	Sensitivity	Specificity	Accuracy
Sopharak et al. [53]	Median filter, contrast enhancement, Shade Correction, and extended minima transform	Patient data	Green channel	81.61%	99.9%	99.98%

Krishna et al. [54]	Ensemble-based microaneurysms detector, Walter Klein, and CLACHE	Messidor	Gray scale	—	—	—

Roy et al. [55]	Canny edge detection, morphological reconstruction	DIARETDB1	Green channel	89.5%	82.1%	—

Adal et al. [56]	Contrast enhancement technique, Hessian-based candidate selection algorithm, and SVM classifier	ROC	Green channel	—	44.64%	—

Datta et al. [57]	Contrast Limited Adaptive Histogram Equalization, median filter, and Image Catenation	Private data	Green channel	—	82.64%	99.98%

Giancardo et al. [58]	Radon cliff operator	ROC	Gray scale	41%	—	—

Ding and Ma [59]	Dynamic multiparameter template (DMPT) matching scheme	ROC		96%	—	—

ROC: retinopathy online challenge.

**Table 7:** Different methods for detection of hemorrhages.

Algorithm	Image processing techniques	Database	Color space	Sensitivity	Specificity	Accuracy
Hatanaka et al. [70]	Gamma correction, density analysis	Private data	HSV	80%	88%	—

Tang et al. [71]	Splat feature, filter approach, and wrapper approach	Messidor	Gray scale	—	—	0.96 AUC at splat level and 0.87 at image Level

Saleh and Eswaran [72]	H-maxima transformation and multilevel Thresholding	Private data	Green channel	84.31% and 87.53%	93.63% and 95.08%	—

Lachure et al. [73]	SVM, KNN classifier	Messidor, DB-rect	HIS	90%	100%	—

Sudha and Thirupurasundari [74]	Median filter, adaptive histogram, and KNN Classifier	Messidor	Gray scale	100%	90%	—

Sharma et al. [75]	Dynamic thresholding	DIARETDB1	Green channel	90%	—	—

Akram et al. [76]	Gaussian Mixture Model, Filter Bank	DRIVE, STARE DIARETDB, Messidor	Green channel	97.83%	98.36%	98.12%

Ashraf et al. [77]	Local Binary Pattern (LBP), SVM	DIARETDB1	Green channel	87.48%	85.99%	86.15%

**Table 8:** Different methods for detection of blood vessels.

Algorithm	Image processing techniques	Database	Color space	Sensitivity	Specificity	Accuracy
Bhatia et al. [28]	Gaussian filter, Canny edge detection, morphological operations, and Otsu thresholding	DRIVE STARE	Green channel	70.31%	97.35%	95.23%

Fraz et al. [78]	Ensemble system of bagged and boosted decision trees, Gabor filter, and morphological transformation	STARE DRIVE CHASE_DB1	Green channel	0.75 0.74 0.72	0.97 0.98 0.97	0.95 0.94 0.94

Nguyen et al. [79]	Vessel segmentation based on the line detectors at varying scale	STARE DRIVE	Green channel	—	—	0.93 Acc 0.94 Acc

Fraz et al. [80]	Multiscale line detection method, Gabor filter	DRIVE STARE Messidor	Gray scale	0.73 0.73 0.77	0.97 0.97 0.98	0.94 0.95 0.96

Yin et al. [81]	Spectral clustering technique based on morphological features, Hessian matrix	DRIVE REVIEW	Green channel	—	—	Above 94%

Vega et al. [82]	Lattice Neural Networks with Dendritic Processing	STARE	Green channel	—	—	99.8%

Marín et al. [83]	Neural Network	DRIVE STARE	Green channel	—	—	0.95 0.97

Hou [84]	Multidirectional morphological top-hat transform, rotating structuring element	DRIVE STARE	Green channel	0.73 0.73	0.96 0.96	0.94 0.93

Shami et al. [85]	Morphological operations	Nikookari	Green channel	85.82%	99.98%	—
